# Capacity of Understanding the Future Approaches in Cancer Treatment by Multiple Models of Artificial Intelligence

**DOI:** 10.1007/s13187-025-02706-y

**Published:** 2025-08-15

**Authors:** Hong Xu, Chengyuan Yang, Xiao-yang Hu, Weikuan Gu

**Affiliations:** 1 Heilongjiang Academy of Traditional Chinese Medicine, Sanfu Road 142, Xiangfang District, Harbin, Heilongjiang 150040 People’s Republic of China; 2 Department of Orthopedic Surgery and BME, College of Medicine, University of Tennessee Health Science Center, Memphis, TN 38163 USA; 3 Basic Medical College of Heilongjiang University of Chinese Medicine, Harbin, Heilongjiang, 150040 China; 4 Research Lt. Col. Luke Weathers, Jr. VA Medical Center, 116 N Pauline St, Memphis, TN 38105 USA; 5 Department of Pharmaceutical Sciences, University of Tennessee Health Science Center, 881 Madison Ave, Memphis, TN 38163 USA

**Keywords:** AI, Breast cancer, ChatGPT, DeepSeek, Chondrosarcoma, Drug, Education, Treatment

## Abstract

Artificial intelligence (AI) has emerged as a popular tool in education for disease treatment, not only for patients but also for physicians and scientists. We aimed to explore the educational values of different AI models in future disease treatment by providing them with real-world obstacles in cancer treatment for the most serious types of breast cancer and chondrosarcoma. We first asked seven large AI models to predict the future treatment approaches that would lead to a better outcome for triple-negative breast cancer (TNBC) and dedifferentiated chondrosarcoma (DDCS). We then requested each model to select the best one and provide supporting evidence. Next, the models were requested to provide a plan or clinical trial to test the treatment approach. Our test obtained ten treatment approaches for TNBC and DDCS from each of the seven models. Together, a total of 18 different unique approaches were suggested for TNBC and 34 for DDCS. Modified and/or extended usage of antibody-drug conjugates are predominantly selected by models as the best approach for TNBC. Combined immune checkpoint inhibition usage and isocitrate dehydrogenase (IDH) inhibitors were favored by models for DDCS. Specialized CAR-T cell therapy and clustered regularly interspaced short palindromic repeats (CRISPR)-based gene editing were selected by majority of AI models as high risk and high reward approaches. Our study indicated that most AI models are capable of keeping up with updated cancer research. However, for patients and physicians, consultation of multiple AI models may gain a better understanding of the pros and cons of a variety of approaches for cancer treatment.

**Supplementary Information:**

The online version contains supplementary material available at https://doi.org/10.1007/s13187-025-02706-y.

## Introduction

Recently, several studies have shown AI can be a potentially impactful education tool in cancer treatment for patients as well as for physicians and researchers [1–6]. AI-assisted areas of education include hypothesis generation in addressing key challenges in the early diagnosis of cancer [2], exploration of the medical applications of AI, including early cancer detection through pathological and imaging analysis, risk stratification, patient triage, and the development of personalized prevention approaches [3]. How to bridge the gap between technological research advancements and their real-world AI-focused clinical applications in cancer care is a critical question [4–6].

With the release of a large number of large language models (LLM), some critical questions have arisen for the public. For example, DeepSeek has been widely used in medical research and has become increasingly relevant. One question is whether this cost-free AI model should be used exclusively. There are reports on the comparisons of applications in the medical field between ChatGPT, DeepSeek, and other AI models [7–10]. While research finds that DeepSeek-V3 provides more suitable responses to patient inquiries [7], another study indicates that integrating the strengths of both models could optimize patient education outcomes [8]. In another study, the authors reported that the domain-specific trained AI tool and ChatGPT-o1 demonstrated superior accuracy [9]. Most importantly, both DeepSeek and ChatGPT have new versions, and several other AI models have been developed and joined the competition, such as Grok3, Claude, Gemini, and Google Copilot [11, 12]. Their performance in other fields has been only initially investigated [11–13].

However, the majority of comparisons of these models have been conducted on the test of common knowledge and data analysis [11]. Their ability to solve real challenges in the medical field has not been tested. In this study, we provide current medical real-world obstacles in cancer treatment to AI models to challenge them for answers. One is the treatment of triple-negative breast cancer (TNBC), the type with the most difficult treatment option in the most common cancer [12]. The other is dedifferentiated chondrosarcoma (DDCS), a cancer type difficult to treat in rare cancer of chondrosarcoma [13]. Our objective is to have a preliminary evaluation of the capability of assisting clinical research and education of 6 AI models, ChatGPT, DeepSeek, Grok 3, Claude, Gemini, and Copilot, in solving real challenges in the real world of cancer treatment.

## Materials and Methods

### AI Databases

We initially included six AI models. They are ChatGPT, DeepSeek, Grok3, Claude, Gemini, and Copilot, which are provided by our institute, the University of Tennessee Health Science Center. For ChatGPT, we included versions 4o and 4.5. Version 4o has been in public for more than 1 year. It is expected that it has made considerable improvement through testing and training by users. Version 4.5 added additional functions, but it is relatively new. For DeepSeek, we used version 3 (R1). For Claude, we used the updated version 4. For Gemini, we used the 2.5 pro. All the tests are performed at the Google Chrome site.

### Request AI Models to Predict Future Treatment that Achieve the Best Outcomes

To each AI model, we first made sure it knows that the most challenging for the treatment of breast cancer is the TNBC (Supplementary Materials). We then asked whether the model knew the reason. When these answers are all positive, we asked the AI model to predict the future treatment approaches (maximum of 10) that will lead to a better outcome for TNBC. We next ask the AI model to select the best one among the approaches that it provided. For each approach, we ask the AI model to provide the title, a brief description in one paragraph, and the key words. Similar questions were asked to AI models for the DDCS.

### Study Plans for the Best Approach from Each AI Model

For the approaches from AI models, we requested each AI model to select the best one. We then asked the reason for the selection and supporting evidence. For the selected approach, we asked the model to provide an experimental plan to test the approach for the best outcomes. For each plan, we asked the model to provide rationale, study design, expected outcomes, potential pitfalls or limitations, and alternative approaches.

### Similarity Comparison for the Future Treatments Provided by AI Models

First, to the set of treatment approaches provided by every AI model, we assigned the approaches from AI models into a number instead of the AI name. In this way, the ten AI models are represented by the numbers from model #1 to model #10. We next collected all these sets of approaches together into one file. We then asked each AI model to compare the similarities among these ten sets of approaches. Based on suggestions from ChatGPT 4o, the similarity was divided into five scores. They are (5) identical or nearly identical, (4) highly similar, (3) moderately similar, (2) slightly similar, and (1) distinct or unrelated.

### Ranking the Best Future Treatments Provided by AI Models

We next asked each AI model to rank the best approaches among the AI models. Because we asked each AI model to select the one best approach from its approaches, we were able to put the total of ten approaches from the ten models into one file. We next provided these ten approaches to each AI model and asked them to rank these approaches. The rankings are based on the 5-score system for each of the five aspects. The five aspects are Clinical Relevance, Personalization, Innovation & Scientific Basis, Feasibility & Safety, and Evidence Support.

### Ranking the Best Study Plan for the Best Approaches Provided by AI Models

For the components of every ten experimental plans, we also numbered them and then asked each AI model to evaluate them. At the end, we asked each AI model to rank the ten experimental plans. The criteria for the evaluation are (1) evaluating the rationale for whether scientific justification and unmet need, (2) methodological rigor and appropriateness for study design, (3) clinical/scientific significance and testability for the expected outcomes, (4) anticipation of limitations or risks for potential pitfalls, and (5) flexibility and creativity in problem-solving for alternative approaches.

### Selection of Approaches for the Future Potential Breakthrough

In order to explore whether AI models can assist in selection approaches that are high risk but potentially make breakthroughs in cancer treatment, we asked each AI model to select one approach that is high risk but may make a breakthrough to the treatment of cancer if successful, from the seven sets of proposed future approaches for each of the diseases, TNBC and DDCS. The selected approaches were then compared, and the supporting materials were reviewed.

## Results

### Predicted Future Treatment that Achieves the Best Outcomes

A total of 18 future approaches for TNBC were proposed by 7 AI models (Supplementary Table 1). There are high similarities in the approaches among AI models (Supplementary Materials Test 1). A total of 34 future approaches for DDCS) were proposed by 7 AI models (Supplementary Table 2). Two approaches, epigenetic therapy and immunotherapy, were proposed by all 7 models (Supplementary Materials Test 2).

### Similarities in the Proposed Approaches Among AI Models

**Fig. 1:**
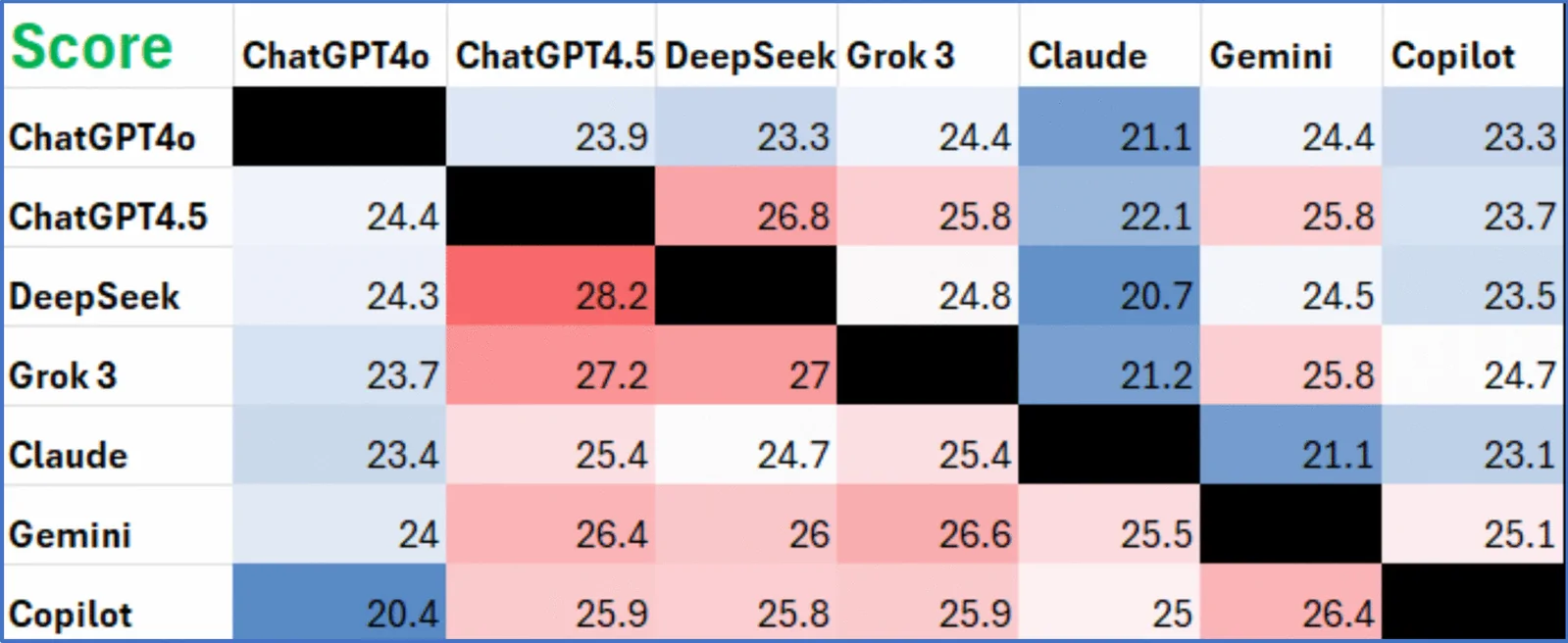
Heatmap of similarity scores of proposed approaches for the treatment of TNBC and DDCS. The numbers of left-low part are for the TNBC, while the right-up part is for the DDCS

Overall, among the total of 18 proposed approaches for TNBC, 9 of them were also proposed to the DDCS. With the nine approaches, epigenetic therapy and immunotherapy are proposed by all AI models for both types of cancer. The majority of proposed approaches for DDCS, 24 of them, are not the same as those for TNBC. Similarity scores for the seven AI models were obtained from six AI models. Overall, similarity scores for the approaches of TNBC are higher than those of DDCS, with every total score for each pair as 25.3 and 23.8, respectively, for TNBC and DDCS, with a *P* value of 0.0005 (Supplementary Table 3 and 4). For TNBC, most of the scores are between 4 and 5 (Supplementary Table 3). The lowest scores for the approaches of both diseases are between the ChatGPT4o and Copilot, with the total scores of 20.4 and 19.3 for TNBC and DDCS, respectively. The highest similarities are for the approaches between DeepSeek and ChatGPT4.5, with scores of 28.2 and 26.6, respectively, for TNBC and DDCS (Figure 1).

### Best Approaches Selected by AI Models

**Fig. 2:**
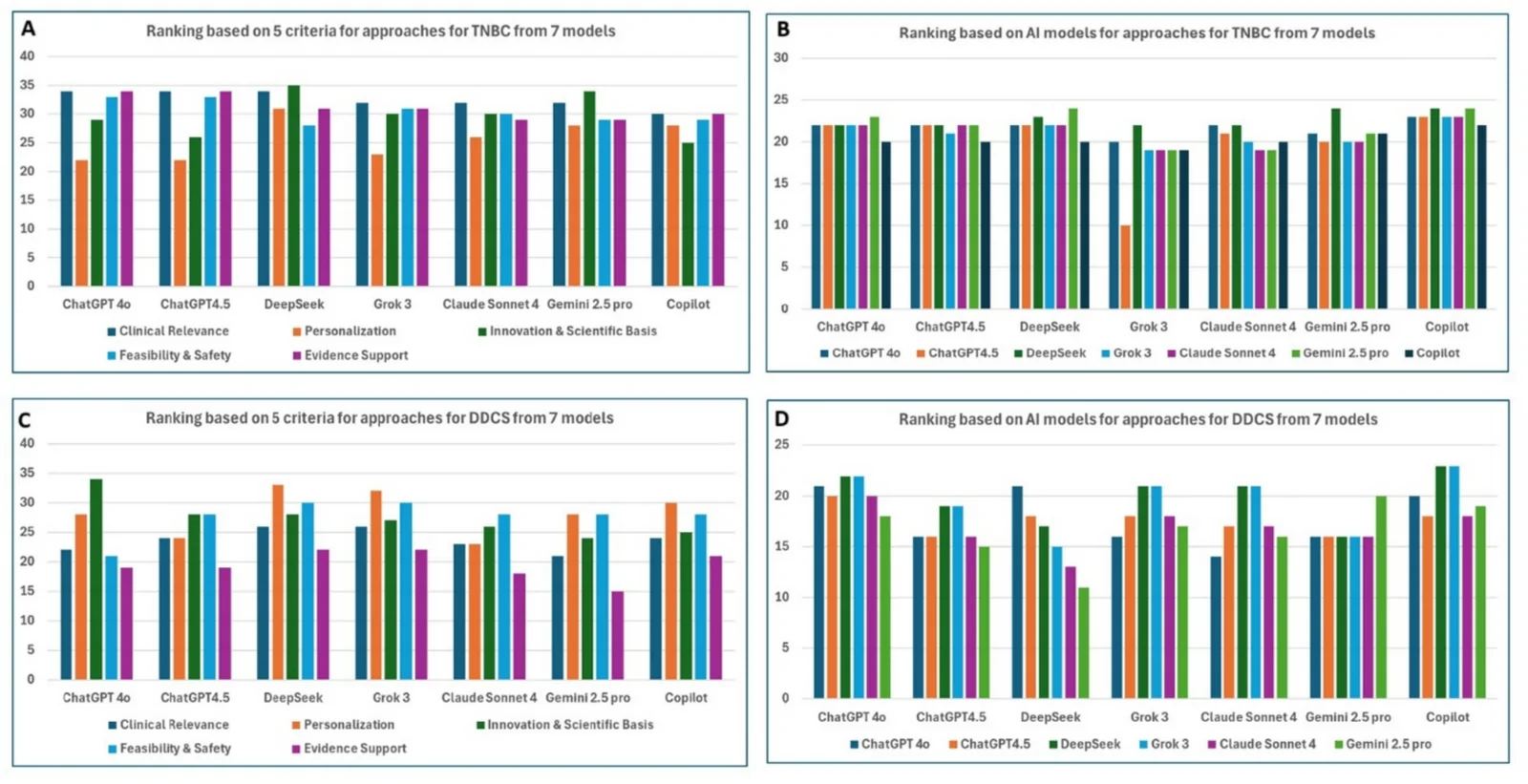
Ranking the approaches provided by AI models. **A** Ranking approaches for TNBC based on the five criteria by the seven AI models. **B** Ranking approaches for TNBC based on the AI models by the seven AI models. **C** Ranking approaches for DDBS based on the five criteria by the seven AI models. **D** Ranking approaches for DDBS based on the AI models by the seven AI models

For the best approaches, 6 AI models selected the antibody-drug conjugates (ADC) except Copilot (Figure 2A). Because they proposed the same approach, the variation is mainly in their utilization of the ADC approach. The scores for the first 6 models range from 140 to 159 (Supplementary Table 5). Ironically, the description of the proposal by ChatGPT4.5 was rated as the lowest, with a total score of 140. The description by DeepSeek received the highest score of 159. ChatGPT4.5 proposed the utilization of Trop-2 targeting (Trop-2 ADC) approach, which is broadly applicable but not highly individualized.

The evaluation of five components by AI models showed a large difference. The largest difference among the five components of evaluation is the Personalization (Figure 2B). All the models were weak in the personalization when they described approaches, with a total score of 180, while the total score for clinical relevance was 228. *P* values for the *T* test for comparison of the scores between these two components resulted in 0.005.

The selected most favorable approaches for the DDCS among the seven AI models are much more diverse than those for TNCB. The total scores for the description of these approaches are much lower than those for TNCB, ranging from 116 to 139 (Supplementary Table 6) (Figure 2C, D). The description of immunotherapy and immune checkpoint inhibitors by Claude Sonnet 4 received the lowest score, while the description of IDH1/2 inhibitors (e.g., Ivosidenib) by DeepSeek received the highest score.

The scores for the 5 components of the approaches range from 136 to 198. Unlike the components for the approaches of TNBC, the component with the lowest score is the description of Evidence Support, a clear indication of lack of knowledge for the DDCS which is a rare type of cancer. The highest score is Personalization, with every score from all AI models higher than that for Evidence Support. Statistical analysis with *T* test showed a significant difference, with *P* value of 0.001 (Supplementary Table 7).

### 4. Experimental Plans to Achieve the Best Treatment

**Fig. 3:**
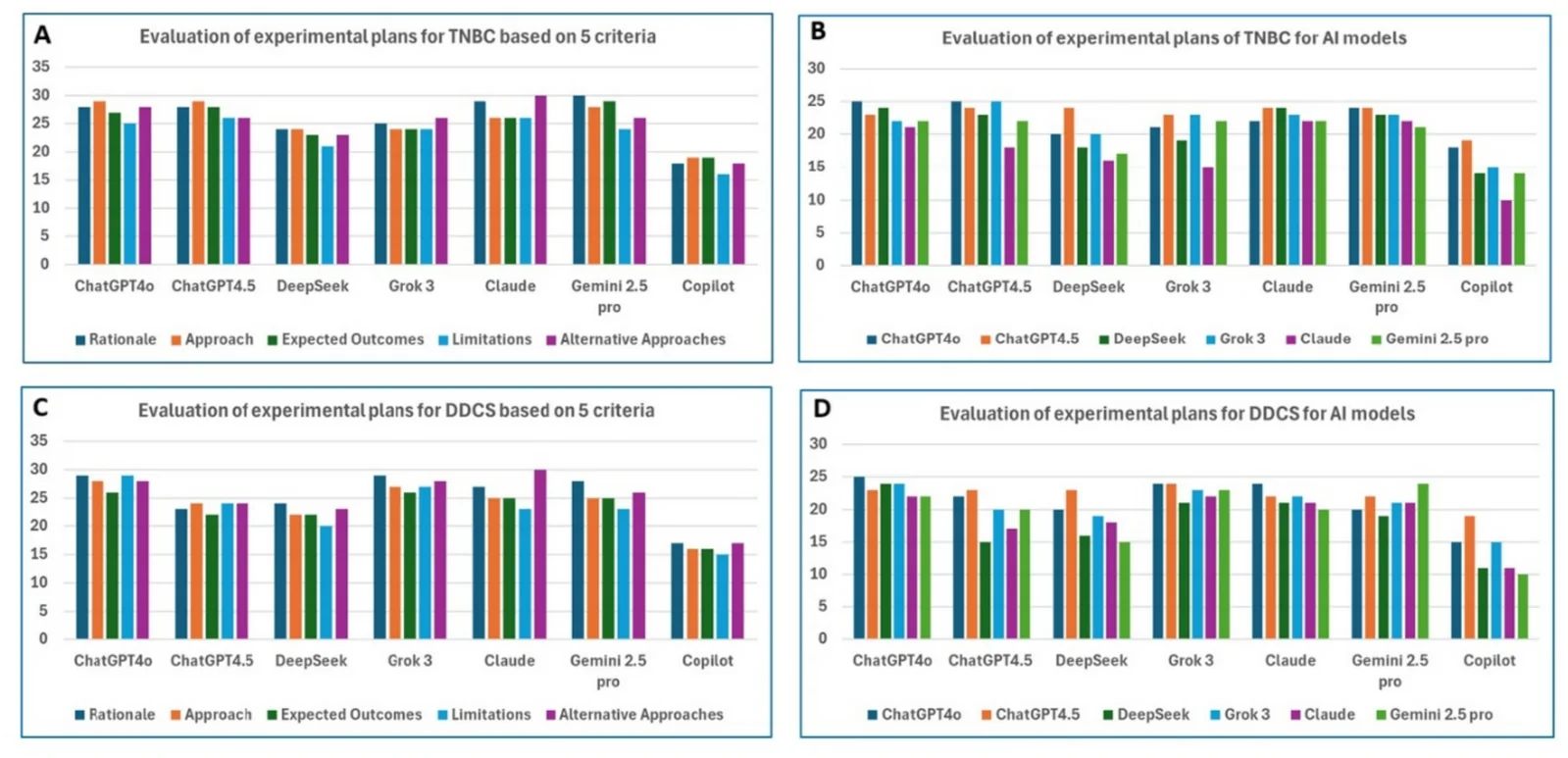
Ranking the experimental plans provided by AI models. **A** Ranking experimental plans for TNBC based on the five components by the seven AI models. **B** Ranking experimental plans for TNBC based on the AI models by the seven AI models. **C** Ranking experimental plans for DDBS based on the five components by the seven AI models. **D** Ranking experimental plans for DDBS based on the AI models by the seven AI models

For most components of the experimental plans of these AI models, the scores are between 4 and 5. The 4 AI models, ChatGPT4o, ChatGPT4.5, Claude Sonnet 4, and Gemini 2.5 pro that described the experimental plans for antibody-drug conjugates (ADCs), received the same high score of 137 (Supplementary Table 8). The high scores for these AI models are the result of their combining innovative targeted therapies (ADCs) with immune modulation, with careful clinical trial design, biomarker integration, and alternative strategies. The experimental plan for the Immunotherapy Enhancements from Copilot received a score of 90. The scores of every 6 AI models are all significantly higher than those of the Copilot, with *P* values less than 0.001 (Figure 3A, B). When examining the scores based on the components of the experimental plans, we found less significant differences, with scores ranging from 162 (Limitations) to 182 (Rationale). However, the scores of four components except the Expected Outcomes are all shown to be statistically significantly higher than those of the Limitations (Supplementary Table 9).

The ranges of scores for the experimental plans for DDCS are much wider than those for the TNBC, from as low as 81 to as high as 140 (Supplementary Table 10). Statistical analysis indicated that all the scores of other AI models are higher than that of Copilot, showing the advantages of large AI models (Figure 3C, D). Although the difference among components of the experimental plans is less than that of the TNBC, ranging from 161 to 177, the components with the lowest and highest scores are the same, with the Limitation as the lowest and the Rationale as the highest. Among the different components, the difference between the Limitation and the Rationale reached a statistically significant level, with a *P* value of 0.034.

### The High Risk and Potentially Breakthrough Approaches

When all the seven sets of the proposed approaches for TNBC and DDCS were provided to each of the AI models and asked them to select one approach in the treatment of TNBC and DDCS, we received answers from six models except Copilot.

Unlike for the selection of the best future approaches, AI selected the approaches showing promises but not having sufficient evidence to support their immediate clinical application. As CAR-T recognizes specific antigens on tumor cells (e.g., CD19 in B-cell malignancies), and the CRISPR-Cas9 system (or variants) is able to cut DNA at targeted sites and induce edits through cellular repair mechanisms, both show the potential of breakthroughs in cancer treatment, while clinical evidence has not yet been confirmed.

## Discussion

Our test indicated that these large AI models follow up the updated information in the medical field and are able to answer questions in depth [14, 15]. The answers from AI models are very intuitive and educational. ADC were not only proposed by all AI models but also were selected as the best approach by the six major AI models [16].

AI models are also able to pick up new ideals for the users. PI3K/AKT/mTOR inhibitors for the future treatment of TNBC were proposed by six models. The PI3K/mTOR pathway is activated in many chondrosarcomas, leading to growth and chemotherapy resistance [17]. Inhibitor for the PI3K/mTOR is presumably inhibiting the cancer cells [18]. AI models are able to provide insight into rare diseases for knowledge and for education. DDCS, as a rare type of cancer, has less developed treatment options [19, 20].

While ADC was selected by six models as the best approach for the treatment of TBC, Copilot proposed an approach of Immunotherapy Enhancements by the utilization of immune checkpoint inhibitors like pembrolizumab [21]. AI models have considerable understanding and knowledge of the novel technologies in the biomedical field. CRISPR-based gene editing and CAR-T cell therapy were also selected as high-risk and high-reward approaches [22–25].

Overall, while outputs from ChatGPT4o and ChatGPT4.5 did not show high similarity, ChatGPT4.5 and DeepSeek showed high similarity in providing information for this study. For the two sets of ten future approaches for TNBC and DDCS, they achieved high similarity scores for both in all pairwise comparisons (Figure 1).

While AI models provided quick and complete updated information on these two types of cancer, AI has considerable limitations including the lack of clinical judgment and ethical considerations [26]. Their output is only based on their training but lacks population specificity. Models trained differently may have model-based bias. In addition, this study is only based on the output of one time; AI models may learn from multiple questions and tests. It is interesting that the scores for the components of the experimental plans, in both cases for the TNBC and DDCS, showed that all AI models did best in the rationale and worst in limitations. This phenomenon may reflect the human nature of good reason for studying but not well on identifying limitations or fear of criticism.

## Conclusion

AI models demonstrate their capability to serve as an educational tool for physicians, patients, and scientists by processing large quantities of data for its output educational materials, which would be impossible for a human to do manually. The values of AI models in classrooms and as teaching materials for the public need to be tested comprehensively in the future.

## Supplementary Information

Below is the link to the electronic supplementary material.Supplementary file1 (DOCX 450 KB)Supplementary file2 (DOCX 88.9 KB)

## Data Availability

All data have been presented in the manuscript and supplemental materials are presented in the supplementary table and available for the public.
