# Supplementary material for: Capacity of Understanding the Future Approaches in Cancer Treatment by Multiple Models of Artificial Intelligence
Source: J Cancer Educ. 2025 Aug 15;41(4):663–9. doi: 10.1007/s13187-025-02706-y (PMC13525040; doi:10.1007/s13187-025-02706-y)
Supplement: Supplementary file 2 [file 13187_2025_2706_MOESM2_ESM.docx]

**Supplementary Table 1. Most favorite future treatment to achieve best outcomes of Triple-Negative Breast Cancer provided by 7 AI models.**

| **Therapeutic Strategy** | ChatGPT 4o | ChatGPT4.5 | DeepSeek | Grok 3 | Claude Sonnet 4 | Gemini 2.5 pro | Copilot |
| --- | --- | --- | --- | --- | --- | --- | --- |
| ****Checkpoint Inhibitors (PD-1/PD-L1, CTLA-4) + combinations (e.g. chemo, CTLA-4)*** | **√** | **√** | **√** | **√** | **√** | **√** | **√** |
| ***PARP inhibitors (BRCA-mutated + BRCAness + combos)*** | **√** | **√** | **√** | **√** | **√** | **√** | **√** |
| ***Antibody-Drug Conjugates (Trop-2, LIV-1, etc.)*** | **√** | **√** | **√** | **√** | **√** | **√** | **√** |
| ***PI3K/AKT/mTOR inhibitors*** | **√** | **√** | **√** | **√** |  | **√** | **√** |
| **Androgen receptor targeting (LAR subtype)** |  | **√** | **√** | **√** |  | **√** |  |
| **Epigenetic therapies (HDAC, DNA methylation)** | **√** | **√** | **√** | **√** | **√** | **√** | **√** |
| **Cancer vaccines (neoantigen-based)** | **√** | **√** | **√** |  |  | **√** |  |
| ***CAR-T or cellular therapies (CAR-T, TILs)*** |  | **√** | **√** |  | **√** | **√** | **√** |
| ***Tumor microenvironment targeting (stroma, TME, angiogenesis)*** |  | **√** |  | **√** | **√** |  | **√** |
| ***Metabolic targeting (glycolysis, glutamine)*** |  |  | **√** | **√** | **√** | **√** | **√** |
| Nanotechnology/nanoparticle delivery | **√** |  | **√** | **√** |  |  |  |
| ***Synthetic lethality beyond PARP*** |  | **√** |  | **√** | **√** |  |  |
| Gene editing (CRISPR) | **√** |  | **√** |  |  |  |  |
| AI-driven precision medicine, multi-omics, liquid biopsy |  |  |  | **√** | **√** | **√** |  |
| Neoadjuvant chemo optimization |  |  |  |  |  |  | **√** |
| Targeting cancer stem cells | **√** |  |  |  |  |  |  |
| ***Oncolytic virus therapy*** | **√** |  |  |  |  |  |  |
| ***Personalized Medicine & Biomarkers*** |  |  |  |  |  |  | **√** |

- *Italic indicates the same as that for DDCS.

Supplementary Table 2. Most favorite future treatment to achieve best outcomes for the Dedifferentiated Chondrosarcoma provided by AI models.

| **Therapeutic Strategy** | ChatGPT 4o | ChatGPT4.5 | DeepSeek | Grok 3 | Claude Sonnet 4 | Gemini 2.5 pro | Copilot |
| --- | --- | --- | --- | --- | --- | --- | --- |
| **Epigenetic therapy (EZH2, HDAC, DNMT)** | **√** | **√** | **√** | **√** | **√** | **√** | **√** |
| **Anti-angiogenic / VEGF inhibitors** | **√** | **√** |  | **√** |  | **√** |  |
| **CDK4/6 inhibitors / cell cycle** |  | **√** | **√** |  | **√** | **√** |  |
| **Oncolytic virus therapy** |  | **√** |  |  | **√** |  |  |
| **Nanoparticle / novel delivery** | **√** |  |  | **√** | **√** |  | **√** |
| **PI3K/mTOR pathway inhibitors** |  | **√** |  | **√** |  |  |  |
| **MDM2-p53 targeting** | **√** |  |  |  |  | **√** |  |
| **Synthetic lethality / DNA repair (PARP)** | **-** | **√** | **√** |  | **√** |  |  |
| **Death receptor agonists (e.g., DR5)** |  |  |  | **√** |  | **√** |  |
| **Hedgehog pathway inhibitors** |  |  |  |  |  | **√** |  |
| **Radiotherapy (proton / advanced RT)** |  |  |  |  |  | **√** | **√** |
| **Tumor microenvironment modulation** | **√** |  |  |  | **√** |  | **√** |
| **Metabolic targeting (glutaminase etc.)** | **√** |  |  |  | **√** |  | **√** |
| **Autophagy modulation** |  |  |  |  | **√** |  |  |
| **Targeted therapy against IDH1/IDH2 mutations** | **√** | **√** | **√** | **√** |  | **√** | **√** |
| **Immunotherapy (e.g., checkpoint inhibitors + oncolytic viruses** | **√** | **√** | **√** | **√** | **√** | **√** | **√** |
| **Patient-specific neoantigen vaccines** | **√** |  |  |  |  |  |  |
| **CAR-T or CAR-NK cells targeting novel surface antigens (e.g., GD2** | **√** | **√** | **√** | **√** | **√** |  | **√** |
| **Multi-omics-driven personalized therapy** |  | **√** |  |  |  |  |  |
| **PDGFRB-Targeted Therapy** |  |  | **√** |  |  |  |  |
| **Antibody-Drug Conjugates (ADCs)** |  |  | **√** |  |  |  |  |
| **TERT-Targeted Therapy** |  |  | **√** |  |  |  |  |
| **3D-Printed Surgical Guides + Proton Therapy** |  |  | **√** |  |  |  |  |
| **Targeted Combination Therapies** |  |  |  | **√** |  |  |  |
| **Hypoxia-Activated Prodrugs** |  |  |  | **√** |  |  |  |
| **Adoptive Cell Therapy** |  |  |  |  |  | **√** |  |
| **Combination Chemotherapy Optimization** |  |  |  |  |  |  | **√** |
| **Personalized Medicine & Biomarkers** |  |  |  |  |  |  | **√** |

Supplementary Table 3. Similarity scores for approaches of TNBC.

| ChatGPT4o | **ChatGPT4o** | **ChatGPT4.5** | **DeepSeek** | **Grok 3** | **Claude** | **Gemini** | **Copilot** |
| --- | --- | --- | --- | --- | --- | --- | --- |
| **ChatGPT4o** | 5 | 4 | 4 | 4 | 4 | 4 | 3 |
| **ChatGPT4.5** | 4 | 5 | 5 | 5 | 4 | 4 | 4 |
| **DeepSeek** | 4 | 5 | 5 | 5 | 4 | 4 | 4 |
| **Grok 3** | 4 | 5 | 5 | 5 | 4 | 4 | 4 |
| **Claude** | 4 | 4 | 4 | 4 | 5 | 4 | 4 |
| **Gemini** | 4 | 4 | 4 | 4 | 4 | 5 | 4 |
| **Copilot** | 3 | 4 | 4 | 4 | 4 | 4 | 5 |
|  |  |  |  |  |  |  |  |
|  |  |  |  |  |  |  |  |
| ChatGPT4.5 | **ChatGPT4o** | **ChatGPT4.5** | **DeepSeek** | **Grok 3** | **Claude** | **Gemini** | **Copilot** |
| **ChatGPT4o** | — | 4.5 | 4.5 | 4.5 | 4.5 | 4.5 | 4.5 |
| **ChatGPT4.5** | 4.5 | — | 5 | 5 | 5 | 5 | 5 |
| **DeepSeek** | 4.5 | 5 | — | 5 | 5 | 5 | 5 |
| **Grok 3** | 4.5 | 5 | 5 | — | 5 | 5 | 5 |
| **Claude** | 4.5 | 5 | 5 | 5 | — | 5 | 5 |
| **Gemini** | 4.5 | 5 | 5 | 5 | 5 | — | 5 |
| **Copilot** | 4.5 | 5 | 5 | 5 | 5 | 5 | — |
|  |  |  |  |  |  |  |  |
|  |  |  |  |  |  |  |  |
| DeepSeek | **ChatGPT4o** | **ChatGPT4.5** | **DeepSeek** | **Grok 3** | **Claude** | **Gemini** | **Copilot** |
| **ChatGPT4o** | — | 4.5 | 4.3 | 4 | 3.8 | 4.2 | 4 |
| **ChatGPT4.5** | 4.5 | — | 4.8 | 4.7 | 4.2 | 4.7 | 4.5 |
| **DeepSeek** | 4.3 | 4.8 | — | 4.9 | 4 | 4.8 | 4.7 |
| **Grok 3** | 4 | 4.7 | 4.9 | — | 4.3 | 4.8 | 4.6 |
| **Claude** | 3.8 | 4.2 | 4 | 4.3 | — | 4.1 | 4 |
| **Gemini** | 4.2 | 4.7 | 4.8 | 4.8 | 4.1 | — | 4.9 |
| **Copilot** | 4 | 4.5 | 4.7 | 4.6 | 4 | 4.9 | — |
|  |  |  |  |  |  |  |  |
|  |  |  |  |  |  |  |  |
| Grok 3 | **ChatGPT4o** | **ChatGPT4.5** | **DeepSeek** | **Grok 3** | **Claude** | **Gemini** | **Copilot** |
| **ChatGPT4o** | 5 | 4 | 4 | 4 | 4 | 4 | 3 |
| **ChatGPT4.5** | 4 | 5 | 4 | 4 | 4 | 4 | 4 |
| **DeepSeek** | 4 | 4 | 5 | 4 | 4 | 4 | 4 |
| **Grok 3** | 4 | 4 | 4 | 5 | 4 | 4 | 4 |
| **Claude** | 4 | 4 | 4 | 4 | 5 | 4 | 4 |
| **Gemini** | 4 | 4 | 4 | 4 | 4 | 5 | 4 |
| **Copilot** | 3 | 4 | 4 | 4 | 4 | 4 | 5 |
|  |  |  |  |  |  |  |  |
|  |  |  |  |  |  |  |  |
| **Claude sonnet 4** | **ChatGPT4o** | **ChatGPT4.5** | **DeepSeek** | **Grok 3** | **Claude** | **Gemini** | **Copilot** |
| **ChatGPT4o** | **-** | **4** | **4** | **4** | **4** | **4** | **3** |
| **ChatGPT4.5** | **4** | **-** | **5** | **4** | **4** | **4** | **4** |
| **DeepSeek** | **4** | **5** | **-** | **4** | **4** | **4** | **4** |
| **Grok 3** | **4** | **4** | **4** | **-** | **4** | **4** | **4** |
| **Claude** | **4** | **4** | **4** | **4** | **-** | **4** | **4** |
| **Gemini** | **4** | **4** | **4** | **4** | **4** | **-** | **4** |
| **Copilot** | **3** | **4** | **4** | **4** | **4** | **4** | **-** |
|  |  |  |  |  |  |  |  |
|  |  |  |  |  |  |  |  |
| Gemini 2.5 pro | **ChatGPT4o** | **ChatGPT4.5** | **DeepSeek** | **Grok 3** | **Claude** | **Gemini** | **Copilot** |
| **ChatGPT4o** | **5** | 3.4 | 3.5 | 3.2 | 3.1 | 3.3 | 2.9 |
| **ChatGPT4.5** | 3.4 | **5** | 4.4 | 4.5 | 4.2 | 4.7 | 4.4 |
| **DeepSeek** | 3.5 | 4.4 | **5** | 4.1 | 3.7 | 4.2 | 4.1 |
| **Grok 3** | 3.2 | 4.5 | 4.1 | **5** | 4.1 | 4.8 | 4.3 |
| **Claude** | 3.1 | 4.2 | 3.7 | 4.1 | **5** | 4.4 | 4 |
| **Gemini** | 3.3 | 4.7 | 4.2 | 4.8 | 4.4 | **5** | 4.5 |
| **Copilot** | 2.9 | 4.4 | 4.1 | 4.3 | 4 | 4.5 | **5** |

Supplementary Table 4. Similarity scores for approaches of DDCS

| ChatGPT4o | **ChatGPT4o** | **ChatGPT4.5** | **DeepSeek** | **Grok 3** | **Claude** | **Gemini** | **Copilot** |
| --- | --- | --- | --- | --- | --- | --- | --- |
| **ChatGPT4o** | - | 4 | 4 | 4 | 4 | 4 | 4 |
| **ChatGPT4.5** | 4 | - | 5 | 4 | 4 | 4 | 4 |
| **DeepSeek** | 4 | 5 | - | 4 | 4 | 4 | 4 |
| **Grok 3** | 4 | 4 | 4 | - | 3 | 4 | 4 |
| **Claude** | 4 | 4 | 4 | 3 | - | 4 | 4 |
| **Gemini** | 4 | 4 | 4 | 4 | 4 | - | 4 |
| **Copilot** | 4 | 4 | 4 | 4 | 4 | 4 | - |
|  |  |  |  |  |  |  |  |
|  |  |  |  |  |  |  |  |
| ChatGPT4.5 | **ChatGPT4o** | **ChatGPT4.5** | **DeepSeek** | **Grok 3** | **Claude** | **Gemini** | **Copilot** |
| **ChatGPT4o** | — | 4.5 | 4.5 | 4.5 | 4 | 4.5 | 4.5 |
| **ChatGPT4.5** | 4.5 | — | 5 | 5 | 4.5 | 5 | 5 |
| **DeepSeek** | 4.5 | 5 | — | 5 | 4.5 | 5 | 5 |
| **Grok 3** | 4.5 | 5 | 5 | — | 4.5 | 5 | 5 |
| **Claude** | 4 | 4.5 | 4.5 | 4.5 | — | 4.5 | 4.5 |
| **Gemini** | 4.5 | 5 | 5 | 5 | 4.5 | — | 5 |
| **Copilot** | 4.5 | 5 | 5 | 5 | 4.5 | 5 | — |
|  |  |  |  |  |  |  |  |
|  |  |  |  |  |  |  |  |
| DeepSeek | **ChatGPT4o** | **ChatGPT4.5** | **DeepSeek** | **Grok 3** | **Claude** | **Gemini** | **Copilot** |
| **ChatGPT4o** | — | 4.2 | 4 | 3.8 | 3.5 | 4 | 3.8 |
| **ChatGPT4.5** | 4.2 | — | 4.8 | 4.5 | 3.8 | 4.7 | 4.5 |
| **DeepSeek** | 4 | 4.8 | — | 4.9 | 3.7 | 4.8 | 4.7 |
| **Grok 3** | 3.8 | 4.5 | 4.9 | — | 4 | 4.8 | 4.6 |
| **Claude** | 3.5 | 3.8 | 3.7 | 4 | — | 3.8 | 3.7 |
| **Gemini** | 4 | 4.7 | 4.8 | 4.8 | 3.8 | — | 4.9 |
| **Copilot** | 3.8 | 4.5 | 4.7 | 4.6 | 3.7 | 4.9 | — |
|  |  |  |  |  |  |  |  |
|  |  |  |  |  |  |  |  |
| Grok 3 | **ChatGPT4o** | **ChatGPT4.5** | **DeepSeek** | **Grok 3** | **Claude** | **Gemini** | **Copilot** |
| **ChatGPT4o** | - | 4 | 4 | 4 | 3 | 4 | 3 |
| **ChatGPT4.5** | 4 | - | 4 | 4 | 3 | 4 | 3 |
| **DeepSeek** | 4 | 4 | - | 4 | 3 | 4 | 3 |
| **Grok 3** | 4 | 4 | 4 | - | 3 | 4 | 3 |
| **Claude** | 3 | 3 | 3 | 3 | - | 3 | 3 |
| **Gemini** | 4 | 4 | 4 | 4 | 3 | - | 3 |
| **Copilot** | 3 | 3 | 3 | 3 | 3 | 3 | - |
|  |  |  |  |  |  |  |  |
|  |  |  |  |  |  |  |  |
| **Claude 3.7 Sonnet** | **ChatGPT4o** | **ChatGPT4.5** | **DeepSeek** | **Grok 3** | **Claude** | **Gemini** | **Copilot** |
| **ChatGPT4o** | - | 4.2 | 3.8 | 4.1 | 3.6 | 3.9 | 4 |
| **ChatGPT4.5** | 4.2 | - | 4 | 4.3 | 3.8 | 4.1 | 4.2 |
| **DeepSeek** | 3.8 | 4 | - | 3.9 | 3.5 | 3.7 | 3.8 |
| **Grok 3** | 4.1 | 4.3 | 3.9 | - | 3.7 | 4 | 4.1 |
| **Claude** | 3.6 | 3.8 | 3.5 | 3.7 | - | 3.8 | 3.9 |
| **Gemini** | 3.9 | 4.1 | 3.7 | 4 | 3.8 | - | 4.2 |
| **Copilot** | 4 | 4.2 | 3.8 | 4.1 | 3.9 | 4.2 | - |
|  |  |  |  |  |  |  |  |
|  |  |  |  |  |  |  |  |
| Gemini2.5 pro | **ChatGPT4o** | **ChatGPT4.5** | **DeepSeek** | **Grok 3** | **Claude** | **Gemini** | **Copilot** |
| **ChatGPT4o** | **5** | 3 | 3 | 4 | 3 | 4 | 4 |
| **ChatGPT4.5** | 3 | **5** | 4 | 4 | 3 | 4 | 3 |
| **DeepSeek** | 3 | 4 | **5** | 3 | 2 | 3 | 3 |
| **Grok 3** | 4 | 4 | 3 | **5** | 3 | 4 | 4 |
| **Claude** | 3 | 3 | 2 | 3 | **5** | 2 | 4 |
| **Gemini** | 4 | 4 | 3 | 4 | 2 | **5** | 4 |
| **Copilot** | 4 | 3 | 3 | 4 | 4 | 4 | 5 |

Supplementary Table 5. Ranking the best approach for Triple-Negative Breast Cancer by different AI models.

| Approaches | ChatGPT 4o | ChatGPT4.5 | DeepSeek | Grok 3 | Claude Sonnet 4 | Gemini 2.5 pro | Copilot | ∑ |
| --- | --- | --- | --- | --- | --- | --- | --- | --- |
| Approach 1 | Trop-2-targeted Antibody–Drug Conjugates (ADCs) | | | | | | | 0 |
| Clinical Relevance | 5 | 5 | 5 | 4 | 5 | 5 | 5 | 34 |
| Personalization | 3 | 3 | 3 | 3 | 3 | 3 | 4 | 22 |
| Innovation & Scientific Basis | 4 | 4 | 4 | 4 | 4 | 4 | 5 | 29 |
| Feasibility & Safety | 5 | 5 | 5 | 4 | 5 | 5 | 4 | 33 |
| Evidence Support | 5 | 5 | 5 | 5 | 5 | 4 | 5 | 34 |
| ∑ | 22 | 22 | 22 | 20 | 22 | 21 | 23 | 152 |
| Approach 2 | Sacituzumab govitecan (SG)—a Trop 2 targeted antibody drug conjugate (ADC) | | | | | | | 0 |
| Clinical Relevance | 5 | 5 | 5 | 4 | 5 | 5 | 5 | 34 |
| Personalization | 3 | 3 | 3 | 3 | 3 | 3 | 4 | 22 |
| Innovation & Scientific Basis | 4 | 4 | 4 | 3 | 3 | 3 | 5 | 26 |
| Feasibility & Safety | 5 | 5 | 5 | 4 | 5 | 5 | 4 | 33 |
| Evidence Support | 5 | 5 | 5 | 5 | 5 | 4 | 5 | 34 |
| ∑ | 22 | 22 | 22 | 10 | 21 | 20 | 23 | 140 |
| Approach 3 | Antibody-Drug Conjugates (ADCs) + Immunotherapy (Sacituzumab Govitecan + Pembrolizumab) | | | | | | | 0 |
| Clinical Relevance | 5 | 5 | 5 | 5 | 4 | 5 | 5 | 34 |
| Personalization | 4 | 4 | 4 | 4 | 5 | 5 | 5 | 31 |
| Innovation & Scientific Basis | 5 | 5 | 5 | 5 | 5 | 5 | 5 | 35 |
| Feasibility & Safety | 4 | 4 | 4 | 4 | 4 | 4 | 4 | 28 |
| Evidence Support | 4 | 4 | 5 | 4 | 4 | 5 | 5 | 31 |
| ∑ | 22 | 22 | 23 | 22 | 22 | 24 | 24 | 159 |
| Approach 4 | Antibody-Drug Conjugates (ADCs | | | | | | | 0 |
| Clinical Relevance | 5 | 5 | 5 | 4 | 4 | 4 | 5 | 32 |
| Personalization | 3 | 3 | 3 | 3 | 4 | 3 | 4 | 23 |
| Innovation & Scientific Basis | 4 | 5 | 4 | 4 | 4 | 4 | 5 | 30 |
| Feasibility & Safety | 5 | 4 | 5 | 4 | 4 | 5 | 4 | 31 |
| Evidence Support | 5 | 4 | 5 | 4 | 4 | 4 | 5 | 31 |
| ∑ | 22 | 21 | 22 | 19 | 20 | 20 | 23 | 147 |
| Approach 5 | Antibody-Drug Conjugates (ADCs) | | | | | | | 0 |
| Clinical Relevance | 5 | 5 | 5 | 4 | 4 | 4 | 5 | 32 |
| Personalization | 4 | 4 | 3 | 3 | 4 | 4 | 4 | 26 |
| Innovation & Scientific Basis | 4 | 5 | 4 | 4 | 4 | 4 | 5 | 30 |
| Feasibility & Safety | 5 | 4 | 5 | 4 | 4 | 4 | 4 | 30 |
| Evidence Support | 4 | 4 | 5 | 4 | 3 | 4 | 5 | 29 |
| ∑ | 22 | 22 | 22 | 19 | 19 | 20 | 23 | 147 |
| Approach 6 | Antibody-Drug Conjugates (ADCs) | | | | | | | 0 |
| Clinical Relevance | 5 | 5 | 5 | 4 | 4 | 4 | 5 | 32 |
| Personalization | 4 | 4 | 4 | 3 | 4 | 4 | 5 | 28 |
| Innovation & Scientific Basis | 5 | 5 | 5 | 4 | 5 | 5 | 5 | 34 |
| Feasibility & Safety | 5 | 4 | 5 | 4 | 3 | 4 | 4 | 29 |
| Evidence Support | 4 | 4 | 5 | 4 | 3 | 4 | 5 | 29 |
| ∑ | 23 | 22 | 24 | 19 | 19 | 21 | 24 | 152 |
| Approach 7 | Immunotherapy Enhancements | | | | | | | 0 |
| Clinical Relevance | 4 | 4 | 4 | 4 | 5 | 5 | 4 | 30 |
| Personalization | 4 | 4 | 3 | 4 | 4 | 4 | 5 | 28 |
| Innovation & Scientific Basis | 4 | 4 | 4 | 3 | 3 | 2 | 5 | 25 |
| Feasibility & Safety | 4 | 4 | 4 | 4 | 4 | 5 | 4 | 29 |
| Evidence Support | 4 | 4 | 5 | 4 | 4 | 5 | 4 | 30 |
| ∑ | 20 | 20 | 20 | 19 | 20 | 21 | 22 | 142 |

Supplementary Table 6. Ranking approaches to achieve the best treatments of Dedifferentiated Chondrosarcoma by different AI models.

| Approaches | ChatGPT 4o | ChatGPT4.5 | DeepSeek | Grok 3 | Claude Sonnet 4 | Gemini 2.5 pro | Copilot | ∑ |
| --- | --- | --- | --- | --- | --- | --- | --- | --- |
| Approach 1 | Combination of immune checkpoint inhibition + oncolytic virus therapy | | | | | | |  |
| Clinical Relevance | 4 | 2 | 4 | 3 | 2 | 3 | 4 | 22 |
| Personalization | 5 | 4 | 5 | 3 | 3 | 4 | 4 | 28 |
| Innovation & Scientific Basis | 5 | 5 | 4 | 5 | 5 | 5 | 5 | 34 |
| Feasibility & Safety | 4 | 3 | 4 | 3 | 2 | 2 | 3 | 21 |
| Evidence Support | 3 | 2 | 4 | 2 | 2 | 2 | 4 | 19 |
| ∑ | 21 | 16 | 21 | 16 | 14 | 16 | 20 | 124 |
| Approach 2 | Immune Checkpoint Inhibition (ICI) | | | | | | | 0 |
| Clinical Relevance | 4 | 3 | 3 | 3 | 3 | 4 | 4 | 24 |
| Personalization | 4 | 3 | 4 | 4 | 3 | 3 | 3 | 24 |
| Innovation & Scientific Basis | 4 | 4 | 5 | 4 | 4 | 3 | 4 | 28 |
| Feasibility & Safety | 5 | 4 | 3 | 4 | 4 | 4 | 4 | 28 |
| Evidence Support | 3 | 2 | 3 | 3 | 3 | 2 | 3 | 19 |
| ∑ | 20 | 16 | 18 | 18 | 17 | 16 | 18 | 123 |
| Approach 3 | IDH1/2 Inhibitors (e.g., Ivosidenib) | | | | | | | 0 |
| Clinical Relevance | 4 | 4 | 3 | 4 | 4 | 2 | 5 | 26 |
| Personalization | 5 | 4 | 4 | 5 | 5 | 5 | 5 | 33 |
| Innovation & Scientific Basis | 4 | 4 | 4 | 4 | 4 | 4 | 4 | 28 |
| Feasibility & Safety | 5 | 4 | 4 | 4 | 4 | 4 | 5 | 30 |
| Evidence Support | 4 | 3 | 2 | 4 | 4 | 1 | 4 | 22 |
| ∑ | 22 | 19 | 17 | 21 | 21 | 16 | 23 | 139 |
| Approach 4 | IDH inhibitors | | | | | | | 0 |
| Clinical Relevance | 4 | 4 | 3 | 4 | 4 | 2 | 5 | 26 |
| Personalization | 5 | 4 | 3 | 5 | 5 | 5 | 5 | 32 |
| Innovation & Scientific Basis | 4 | 4 | 3 | 4 | 4 | 4 | 4 | 27 |
| Feasibility & Safety | 5 | 4 | 4 | 4 | 4 | 4 | 5 | 30 |
| Evidence Support | 4 | 3 | 2 | 4 | 4 | 1 | 4 | 22 |
| ∑ | 22 | 19 | 15 | 21 | 21 | 16 | 23 | 137 |
| Approach 5 | Immunotherapy and Immune Checkpoint Inhibitors | | | | | | | 0 |
| Clinical Relevance | 4 | 3 | 2 | 3 | 3 | 4 | 4 | 23 |
| Personalization | 4 | 3 | 3 | 4 | 3 | 3 | 3 | 23 |
| Innovation & Scientific Basis | 4 | 4 | 3 | 4 | 4 | 3 | 4 | 26 |
| Feasibility & Safety | 5 | 4 | 3 | 4 | 4 | 4 | 4 | 28 |
| Evidence Support | 3 | 2 | 2 | 3 | 3 | 2 | 3 | 18 |
| ∑ | 20 | 16 | 13 | 18 | 17 | 16 | 18 | 118 |
| Approach 6 | CDK4/6 inhibitors | | | | | | | 0 |
| Clinical Relevance | 3 | 2 | 2 | 3 | 3 | 4 | 4 | 21 |
| Personalization | 5 | 4 | 2 | 4 | 4 | 5 | 4 | 28 |
| Innovation & Scientific Basis | 4 | 4 | 2 | 4 | 3 | 4 | 3 | 24 |
| Feasibility & Safety | 4 | 3 | 3 | 4 | 4 | 5 | 5 | 28 |
| Evidence Support | 2 | 2 | 2 | 2 | 2 | 2 | 3 | 15 |
| ∑ | 18 | 15 | 11 | 17 | 16 | 20 | 19 | 116 |
| Approach 7 | IDH Inhibitors (Ivosidenib) | | | | | | | 0 |
| Clinical Relevance | 4 | 4 | 1 | 4 | 4 | 2 | 5 | 24 |
| Personalization | 5 | 4 | 1 | 5 | 5 | 5 | 5 | 30 |
| Innovation & Scientific Basis | 4 | 4 | 1 | 4 | 4 | 4 | 4 | 25 |
| Feasibility & Safety | 5 | 4 | 2 | 4 | 4 | 4 | 5 | 28 |
| Evidence Support | 4 | 3 | 1 | 4 | 4 | 1 | 4 | 21 |
| ∑ | 22 | 19 | 6 | 21 | 21 | 16 | 23 | 128 |

Supplementary Table 7. Summary of ranking scores for approaches of TNBC

| **TNBC** | **Clinical Relevance** | **Personalization** | **Innovation & Scientific Basis** | **Feasibility & Safety** | **Evidence Support** | **Summary** | T test |
| --- | --- | --- | --- | --- | --- | --- | --- |
| ChatGPT 4o | 34 | 22 | 29 | 33 | 34 | 152 |  |
| ChatGPT4.5 | 34 | 22 | 26 | 33 | 34 | 149 |  |
| DeepSeek | 34 | 31 | 35 | 28 | 31 | 159 |  |
| Grok 3 | 32 | 23 | 30 | 31 | 31 | 147 |  |
| Claude Sonnet 4 | 32 | 26 | 30 | 30 | 29 | 147 |  |
| Gemini 2.5 pro | 32 | 28 | 34 | 29 | 29 | 152 |  |
| Copilot | 30 | 28 | 25 | 29 | 30 | 142 |  |
|  | 228 | 180 | 209 | 213 | 218 |  | 0.004876 |

Supplementary Table 8. Evaluation of experimental plans to achieve the best treatments of Triple-Negative Breast Cancer by different AI models.

| Treatment | ChatGPT4o | ChatGPT4.5 | DeepSeek | Grok 3 | Claude | Gemini 2.5 pro | Copilot | Summary |
| --- | --- | --- | --- | --- | --- | --- | --- | --- |
| 1 | Trop-2-targeted Antibody–Drug Conjugates (ADCs) | | | | | | | |
| Rationale | 5 | 5 | 5 | 4 | 4 | 5 |  | 28 |
| Approach | 5 | 5 | 5 | 5 | 5 | 4 |  | 29 |
| Expected Outcomes | 5 | 5 | 5 | 4 | 4 | 4 |  | 27 |
| Limitations | 5 | 4 | 4 | 4 | 4 | 4 |  | 25 |
| Alternative Approaches | 5 | 4 | 5 | 5 | 4 | 5 |  | 28 |
| ∑ | 25 | 23 | 24 | 22 | 21 | 22 | 0 | 137 |
| 2 | Sacituzumab govitecan (SG)—a Trop 2 targeted antibody drug conjugate (ADC) | | | | | | | |
| Rationale | 5 | 5 | 5 | 5 | 4 | 4 |  | 28 |
| Approach | 5 | 5 | 5 | 5 | 4 | 5 |  | 29 |
| Expected Outcomes | 5 | 5 | 5 | 5 | 4 | 4 |  | 28 |
| Potential Pitfalls | 5 | 4 | 4 | 5 | 3 | 5 |  | 26 |
| Alternative Approaches | 5 | 5 | 4 | 5 | 3 | 4 |  | 26 |
| ∑ | 25 | 24 | 23 | 25 | 18 | 22 | 0 | 137 |
| 3 | Antibody-Drug Conjugates (ADCs) + Immunotherapy (Sacituzumab Govitecan + Pembrolizumab) | | | | | | | |
| Rationale | 4 | 5 | 4 | 4 | 3 | 4 |  | 24 |
| Approach | 4 | 5 | 4 | 4 | 4 | 3 |  | 24 |
| Expected Outcomes | 4 | 5 | 4 | 4 | 3 | 3 |  | 23 |
| Potential Pitfalls | 4 | 4 | 3 | 4 | 3 | 3 |  | 21 |
| Alternative Approaches | 4 | 5 | 3 | 4 | 3 | 4 |  | 23 |
| ∑ | 20 | 24 | 18 | 20 | 16 | 17 | 0 | 115 |
| 4 | Antibody-Drug Conjugates (ADCs) | | | | | | | |
| Rationale | 5 | 5 | 4 | 4 | 3 | 4 |  | 25 |
| Approach | 4 | 4 | 4 | 5 | 3 | 4 |  | 24 |
| Expected Outcomes | 4 | 5 | 4 | 4 | 3 | 4 |  | 24 |
| Potential Pitfalls | 4 | 4 | 3 | 5 | 3 | 5 |  | 24 |
| Alternative Approaches | 4 | 5 | 4 | 5 | 3 | 5 |  | 26 |
| ∑ | 21 | 23 | 19 | 23 | 15 | 22 | 0 | 123 |
| 5 | Antibody-Drug Conjugates (ADCs) | | | | | | | |
| Rationale | 5 | 5 | 5 | 5 | 4 | 5 |  | 29 |
| Approach | 4 | 5 | 5 | 4 | 4 | 4 |  | 26 |
| Expected Outcomes | 4 | 5 | 5 | 4 | 4 | 4 |  | 26 |
| Potential Pitfalls | 4 | 4 | 4 | 5 | 5 | 4 |  | 26 |
| Alternative Approaches | 5 | 5 | 5 | 5 | 5 | 5 |  | 30 |
| ∑ | 22 | 24 | 24 | 23 | 22 | 22 | 0 | 137 |
| 6 | Antibody-Drug Conjugates (ADCs) | | | | | | | |
| Rationale | 5 | 5 | 5 | 5 | 5 | 5 |  | 30 |
| Approach | 5 | 5 | 5 | 5 | 4 | 4 |  | 28 |
| Expected Outcomes | 5 | 5 | 5 | 5 | 5 | 4 |  | 29 |
| Potential Pitfalls | 4 | 4 | 4 | 4 | 4 | 4 |  | 24 |
| Alternative Approaches | 5 | 5 | 4 | 4 | 4 | 4 |  | 26 |
| ∑ | 24 | 24 | 23 | 23 | 22 | 21 | 0 | 137 |
| 7 | Immunotherapy Enhancements | | | | | | | |
| Rationale | 4 | 4 | 3 | 3 | 2 | 2 |  | 18 |
| Approach | 4 | 4 | 3 | 3 | 2 | 3 |  | 19 |
| Expected Outcomes | 4 | 4 | 3 | 3 | 2 | 3 |  | 19 |
| Potential Pitfalls | 3 | 3 | 2 | 3 | 2 | 3 |  | 16 |
| Alternative Approaches | 3 | 4 | 3 | 3 | 2 | 3 |  | 18 |
| ∑ | 18 | 19 | 14 | 15 | 10 | 14 | 0 | 90 |

Supplementary Table 9. Summary of ranking scores for approaches of DDCS

| **DDCS** | **Clinical Relevance** | **Personalization** | **Innovation & Scientific Basis** | **Feasibility & Safety** | **Evidence Support** | | **Summary** |
| --- | --- | --- | --- | --- | --- | --- | --- |
| ChatGPT 4o | 22 | 28 | 34 | 21 | 19 | 124 | |
| ChatGPT4.5 | 24 | 24 | 28 | 28 | 19 | 123 | |
| DeepSeek | 26 | 33 | 28 | 30 | 22 | 139 | |
| Grok 3 | 26 | 32 | 27 | 30 | 22 | 137 | |
| Claude Sonnet 4 | 23 | 23 | 26 | 28 | 18 | 118 | |
| Gemini 2.5 pro | 21 | 28 | 24 | 28 | 15 | 116 | |
| Copilot | 24 | 30 | 25 | 28 | 21 | 128 | |
|  | 166 | 198 | 192 | 193 | 136 | 0.001155 | |

Supplementary Table 10. Evaluation of experimental plans to achieve the best treatments of Dedifferentiated Chondrosarcoma by different AI models.

| Treatment | ChatGPT4o | ChatGPT4.5 | DeepSeek | Grok 3 | Claude | Gemini 2.5 pro | Copilot | Summary |
| --- | --- | --- | --- | --- | --- | --- | --- | --- |
| 1 | Combination of immune checkpoint inhibition + oncolytic virus therapy | | | | | | |  |
| Rationale | 5 | 5 | 5 | 5 | 4 | 5 |  | 29 |
| Approach | 5 | 4 | 5 | 5 | 5 | 4 |  | 28 |
| Expected Outcomes | 5 | 4 | 5 | 4 | 4 | 4 |  | 26 |
| Limitations | 5 | 5 | 4 | 5 | 5 | 5 |  | 29 |
| Alternative Approaches | 5 | 5 | 5 | 5 | 4 | 4 |  | 28 |
| ∑ | 25 | 23 | 24 | 24 | 22 | 22 | 0 | 140 |
| 2 | Immune Checkpoint Inhibition (ICI) | | | | | | | 0 |
| Rationale | 4 | 5 | 3 | 4 | 3 | 4 |  | 23 |
| Approach | 5 | 4 | 3 | 4 | 4 | 4 |  | 24 |
| Expected Outcomes | 4 | 4 | 3 | 4 | 3 | 4 |  | 22 |
| Potential Pitfalls | 5 | 5 | 3 | 4 | 3 | 4 |  | 24 |
| Alternative Approaches | 4 | 5 | 3 | 4 | 4 | 4 |  | 24 |
| ∑ | 22 | 23 | 15 | 20 | 17 | 20 | 0 | 117 |
| 3 | IDH1/2 Inhibitors (e.g., Ivosidenib) | | | | | | | 0 |
| Rationale | 4 | 5 | 4 | 4 | 4 | 3 |  | 24 |
| Approach | 4 | 5 | 3 | 4 | 4 | 2 |  | 22 |
| Expected Outcomes | 4 | 5 | 3 | 4 | 3 | 3 |  | 22 |
| Potential Pitfalls | 4 | 4 | 3 | 3 | 3 | 3 |  | 20 |
| Alternative Approaches | 4 | 4 | 3 | 4 | 4 | 4 |  | 23 |
| ∑ | 20 | 23 | 16 | 19 | 18 | 15 | 0 | 111 |
| 4 | IDH inhibitors | | | | | | | 0 |
| Rationale | 5 | 5 | 5 | 5 | 5 | 4 |  | 29 |
| Approach | 4 | 5 | 4 | 4 | 5 | 5 |  | 27 |
| Expected Outcomes | 5 | 5 | 4 | 4 | 4 | 4 |  | 26 |
| Potential Pitfalls | 5 | 4 | 4 | 5 | 4 | 5 |  | 27 |
| Alternative Approaches | 5 | 5 | 4 | 5 | 4 | 5 |  | 28 |
| ∑ | 24 | 24 | 21 | 23 | 22 | 23 | 0 | 137 |
| 5 | Immunotherapy and Immune Checkpoint Inhibitors | | | | | | | 0 |
| Rationale | 5 | 5 | 5 | 4 | 4 | 4 |  | 27 |
| Approach | 5 | 4 | 4 | 5 | 4 | 3 |  | 25 |
| Expected Outcomes | 5 | 4 | 4 | 4 | 4 | 4 |  | 25 |
| Potential Pitfalls | 4 | 4 | 3 | 4 | 4 | 4 |  | 23 |
| Alternative Approaches | 5 | 5 | 5 | 5 | 5 | 5 |  | 30 |
| ∑ | 24 | 22 | 21 | 22 | 21 | 20 | 0 | 130 |
| 6 | CDK4/6 inhibitors | | | | | | | 0 |
| Rationale | 4 | 5 | 4 | 5 | 5 | 5 |  | 28 |
| Approach | 4 | 4 | 4 | 4 | 4 | 5 |  | 25 |
| Expected Outcomes | 4 | 4 | 4 | 4 | 4 | 5 |  | 25 |
| Potential Pitfalls | 4 | 4 | 3 | 4 | 4 | 4 |  | 23 |
| Alternative Approaches | 4 | 5 | 4 | 4 | 4 | 5 |  | 26 |
| ∑ | 20 | 22 | 19 | 21 | 21 | 24 | 0 | 127 |
| 7 | IDH Inhibitors (Ivosidenib) | | | | | | | 0 |
| Rationale | 3 | 4 | 3 | 3 | 2 | 2 |  | 17 |
| Approach | 3 | 4 | 2 | 3 | 2 | 2 |  | 16 |
| Expected Outcomes | 3 | 4 | 2 | 3 | 2 | 2 |  | 16 |
| Potential Pitfalls | 3 | 3 | 2 | 3 | 2 | 2 |  | 15 |
| Alternative Approaches | 3 | 4 | 2 | 3 | 3 | 2 |  | 17 |
| ∑ | 15 | 19 | 11 | 15 | 11 | 10 | 0 | 81 |

Supplementary Table 11. Selected high risk and high reward approach for TNBC

| **AI Model** | **Selected high risk and high reward approach for TNBC** |
| --- | --- |
| ChatGPRT4o | CAR-T Cell Therapy (or Cellular Therapies such as CAR-T and TILs) |
| ChatGPT4.5 | CRISPR-based gene editing / gene-editing tools to correct oncogenic drivers or enhance immune recognition of TNBC cells |
| DeepSeek | CRISPR-Based Gene Editing or CAR-T Cell Therapy |
| Grok 3 | CRISPR-based gene editing) |
| Claude Sonnet 4 | CRISPR-based gene editing |
| Gemini 2.5 pro | CRISPR-based gene editing |
| Copilot | - |

Supplementary Table 12. Selected high risk and high reward approach for DDCS

| **AI Model** | **Selected high risk and high reward approach for DDCS** |
| --- | --- |
| ChatGPRT4o | CAR-T Cell Therapy |
| ChatGPT4.5 | CAR-T / CAR-NK / T-cell therapies t |
| DeepSeek | CAR-T Cell Therapy Targeting Novel Surface Antigens |
| Grok 3 | CRISPR-mediated correction of driver mutations |
| Claude Sonnet 4 | CRISPR-mediated correction of driver mutations |
| Gemini 2.5 pro | CAR-T / CAR-NK Cell Therapy |
| Copilot | - |
